# 

**Published:** 1917-05-26

**Authors:** 


### Personalia.

Mr. Kennedy Jones, M.P., has accepted the presidency of Finchley Cottage Hospital.

Stepney Borough Council has appointed Miss Eileen Bertha Hiles-Clements and Miss Dorothy Mitchell as health visitors at salaries of £100 a year, rising to £130.

Camberwell Infirmary.—Dr. C. Rivington, M.R.C.S., L.R.C.P., has been appointed assistant medical superintendent of Camberwell Infirmary for the period of the war, to fill a vacancy in the place of Dr. Stevens, at a salary of £500 per annum.

MR. R. H. LUCKETT, of Erdington, a commercial traveller, left estate valued at £18,162. He bequeathed £500 to the West Bromwich Hospital; £200 each to the West Bromwich District Nursing Association, the Birmingham General Hospital, the Queen's Hospital, Birmingham, and the Birmingham and Midland Eye Hospital, and £100 to the Birmingham and Midland Skin Hospital. The Commercial Travellers' Schools and Benevolent Institution were left £250 each. The residue of the estate will be divided amongst six institutions, including the Birmingham and Midland Children's Hospital.

### Matrons' and Sisters' Appointments.

Clare Hall Sanatorium, South Mimms, Barnet.—Miss Mary Brown has been appointed matron. She was trained at Glasgow, and has been charge nurse and night superintendent at the Fountain Fever Hospital, house matron at the Down School, and matron at the Kilman Convalescent Home.

Colchester Isolation Hospital.—Miss Adah Audrey Patten has been appointed sister. She was trained at the Western Fever Hospital, Fulham, and has since been staff nurse at the Isolation Hospital at Leicester and Wimbledon, charge nurse at the Smith Hospital, Henley-on-Thames, at Folkestone Sanatorium, and at the Borough Hospital, Earlswood, Surrey. She has also done private nursing.

Dorset County Hospital, Dorchester.—Miss Eleanor F. Seymour Mackenzie has been appointed night sister. She was trained at the Birkenhead General Hospital; has been sister at the Auxiliary Military Hospital, Frodsham, Cheshire, at the Bedford County Hospital, and at the Throat Hospital, London.

Harborne Hall Auxiliary Hospital, Birmingham.—Miss Mabel Pineo has been appointed night sister. She was trained at Royal Hants County Hospital, Winchester, and in addition to other posts has been matron of a nursing home at Bournemouth.

Paddington Green Children's Hospital.—Miss Jane Marsh has been appointed sister. She was trained at St. Marylebone Infirmary and Grays Isolation Hospital.

St. James's Infirmary, Balham.—Miss Edith Taylor, Miss Elsie Dalby, and Miss L. A. Flower have been appointed ward sisters. The two former were trained at St. James's Infirmary, and Miss Flower was trained at Booth Hall Infirmary, Manchester, and has been ward sister at Bermondsey Infirmary.

**NOTE.—Particulars of Appointments for insertion in this column will be welcomed.**

### The Book Market.

**LIST OF NEW BOOKS RECEIVED FROM THE FOLLOWING PUBLISHERS:—**

T. Fisher Unwin: "Joan Avenel." By Dora S. Forgan. 6s. net. "Selected Poems of Robert Service." 6d. net.

Gay and Hancock: "Poems of Purpose." By Ella Wheeler Wilcox. 1s. 3d. net.

Wm. Heinemann: "War Shock, the Psycho-Neuroses; Psychology and Treatment." By M. D. Eder. 5s. net.

Longmans, Green and Co.: "The Secretion of Urine." By A. R. Cushny, M.D. 9s. net.

### TO EDUCATE THE EDUCATIONIST.

The Child-Study Society have put forth a carefully considered memorandum followed by a series of practical recommendations addressed successively to the Board of Education, to local authorities, and to teachers of all kinds. From the Board they ask facilities and financial support for research, including the establishment of a special Board, the publication of results, child-study as a special subject in training colleges and summer schools for the purpose, besides the establishment by local authorities of psychological clinics and observation schools. Professor John Adams says in his introduction that our present interest in education is largely the result of the fear of commercial competition, "the schoolmaster won at Sadowa and Gravelotte." Certainly the world has awakened to the value of education as the source of national strength, and realises that those working bees the teachers need to mix the food of the growing colony "with brains, sir!" Even if the Society cannot at present obtain more than a fraction of its demand, it will do much if it raises up a generation of educationists who will not be so incapable of getting knowledge into a child's head through the ear that they must needs, like the mediæval master, whip it in at the other end. The memorandum itself is written for the specialist; it may be welcomed as a promise that more study, both practical and literary, will save teachers from the blind following of a single theorist and from rash experimenting on the child.

RATES OF SUBSCRIPTION: Twelve months, 6/6; Six months, 4s.; three months, 2/-, post free to any address in the United Kingdom. Abroad, Twelve months, 8/8; Six months, 5/-; Three months, 2/6, post free. **THE HOSPITAL "Index" to Volume LXI., October 1916 to March 1917, is now ready, price 2½d. per copy, post free.** Address The Manager, THE HOSPITAL, "The Hospital" Building, 28/29 Southampton Street, Strand, London, W.C. 2.

### "LYSOL" EVANS

(ALSO KNOWN AS "EVANSOL")

**Made with Potashes not Soda.**

***In 4-oz., 8-oz., 16-oz. and 32-oz. Bottles.***

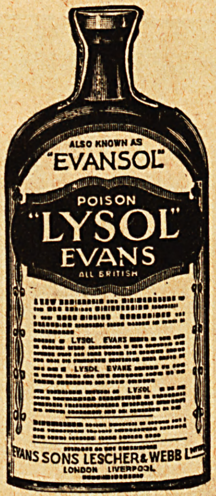

**Gives a Clear Solution.**

***In ½-gall. and 1-gall. Tins.***

Specify "LYSOL" EVANS and ensure a standard article that can be completely relied upon.

*On application we will send Free, a full 4-oz. Sample to Medical Men, Nurses and Institutions.*

EVANS SONS LESCHER & WEBB LIMITED.

60 Bartholomew Close, LONDON. 56 Hanover Street, LIVERPOOL.

A

### A NEW TREATMENT FOR GONORRHŒA.

By CHARLES RUSS, M.B., M.R.C.S.,

*Physician-in-Charge, Electro-Therapeutic Department, Male Lock Hospital, London.*

Demy 8vo, 3s. net; post free, 3s. 4d.

"We have read this book with interest and profit, and cordially recommend it to the profession."—*The Medical Press.*

"This form of treatment merits further attention, as it is both scientific and ingenious."—*British Medical Journal.*

**A New Edition is in preparation.**

London: H. K. LEWIS & CO., Ltd., 136 Gower Street, W.C.

### HUMAN TEMPERAMENTS:

**Studies in Character.**

By Dr. CHARLES MERCIER.

**1/3 net. By Post 1/4.**

"The essays are written in Dr. Mercier's well-known style—they are witty, pithy, and display a wide range of observation and learning. We recommend the brochure cordially to our readers. It is always extremely readable and often very wise."—*The Lancet.*

"Dr. Mercier is a well-known authority on mental diseases, and his practical knowledge assists him in this analysis of the temperaments of the artist, the faddist, the practical man, the envious man, the philosopher, and so on. Dr. Mercier can claim that the reader may find some widening of his knowledge of human nature as the result of these shrewd and experienced studies."—*The Times.*

"It is long since within so small a compass so much help was given to mankind in the pursuit of that 'proper study,' the knowledge of his fellow-man."—*The Hospital.*

*Of all Booksellers, or of*

**The Scientific Press, Ltd.,**

**28/29 Southampton Street, Strand, London, W.C. 2.**

